# Transgenic Plant-Produced Hydrolytic Enzymes and the Potential of Insect Gut-Derived Hydrolases for Biofuels

**DOI:** 10.3389/fpls.2016.00675

**Published:** 2016-05-31

**Authors:** Jonathan D. Willis, Mitra Mazarei, C. Neal Stewart

**Affiliations:** ^1^Department of Plant Sciences, University of TennesseeKnoxville, TN, USA; ^2^Oak Ridge National Laboratory, BioEnergy Science CenterOak Ridge, TN, USA

**Keywords:** lignocellulosic, insect cellulase, cell wall degrading (CWD) enzyme, biofuel, hydrolase, gene expression, enzyme targeting

## Abstract

Various perennial C4 grass species have tremendous potential for use as lignocellulosic biofuel feedstocks. Currently available grasses require costly pre-treatment and exogenous hydrolytic enzyme application to break down complex cell wall polymers into sugars that can then be fermented into ethanol. It has long been hypothesized that engineered feedstock production of cell wall degrading (CWD) enzymes would be an efficient production platform for of exogenous hydrolytic enzymes. Most research has focused on plant overexpression of CWD enzyme-coding genes from free-living bacteria and fungi that naturally break down plant cell walls. Recently, it has been found that insect digestive tracts harbor novel sources of lignocellulolytic biocatalysts that might be exploited for biofuel production. These CWD enzyme genes can be located in the insect genomes or in symbiotic microbes. When CWD genes are transformed into plants, negative pleiotropic effects are possible such as unintended cell wall digestion. The use of codon optimization along with organelle and tissue specific targeting improves CWD enzyme yields. The literature teaches several important lessons on strategic deployment of CWD genes in transgenic plants, which is the focus of this review.

## Introduction

The natural recalcitrance of plant cell walls is a major commercial hurdle for cellulosic biofuel production. Two economic barriers of biofuel production are pretreatment and hydrolytic enzymes (Wyman, 2007; Furtado et al., 2014). It has been hypothesized that using a transgenic plant vehicle for the production of hydrolytic enzymes would improve cellulosic biofuel economics. Thus far, plant species transformed to overproduce hydrolytic enzymes are not bioenergy feedstock crops, but rather, easily transformed species such as *Arabidopsis thaliana, Nicotiana tabacum* (tobacco), and *Oryza sativa* (rice) (Taylor et al., 2008). All of these models are annual plants, and therefore may not be entirely predictive of enzyme activity perennial crops forecasted to be the most useful for cellulosic bioenergy.

Plant cell walls are composed of three major types of polymers: cellulose, hemicelluloses, and lignin. Each of these polymers are cross-linked and/or intertwined with another type to provide stereochemical strength (Cosgrove, 2005). Plant cell walls have successfully been broken down using mechanical and chemical means, however these processes can release metabolites that can inhibit fermentation. Furfural is one such metabolite that inhibits the growth of yeast (Boyer et al., 1992; Zhang K. et al., 2011). A mixture of mechanical, chemical, and enzymatic means might be required to reduce cell wall recalcitrance in lignocellulosic biomass processing. Enzymes represent one of the highest material costs in lignocellulosic processing (Taylor et al., 2008; Klein-Marcuschamer et al., 2012).

Synergistic action of multiple enzymes are required for complete cell wall degradation. Four-to-five classes of enzymes are required to break down cellulose into its smaller cellobiose sub-units (Bhat, 2000; Lopez-Casado et al., 2008), which is, in turn, composed of two glucose units connected by a β-1,4 linkage. The tightly-packed crystalline structure of cellulose prevents enzyme accessibility. Hemicelluloses have variable polymeric structures composed of pentose sugars and a variety of side chains, which necessitates a suite of enzymes for hydrolysis (Saha, 2003; Scheller and Ulvskov, 2010).

Before the advent of rapid DNA sequencing technologies, glycosyl hydrolase (GH) enzymes were categorized based on their substrate specificity. Substrate specificity categorization was inadequate with discovery of enzymes with multiple catalytic domains and/or multiple substrate adherence (Henrissat, 1991). The previous substrate specificity classification system was updated and GHs were re-grouped into families (Henrissat and Bairoch, 1993, 1996). The CAZy database (CAZy.org) provides tools for classification and defining novel cellulolytic enzymes (Lombard et al., 2014).

Enzymes used for industrial cell wall hydrolysis are prepared from mesophilic fungi and bacteria. Optimal enzyme activity in such cases is typically 50°C and a pH range of 5.0–7.0 (Bruce and Palfreyman, 1998; Bhalla et al., 2013). Enzymatic degradation of cellulose to glucose requires three enzyme classes: endo-glucanases, cellobiohydrolases, and β-glucosidases (Box 1). Endo-glucanases (EG) act randomly on internal portions of the cellulose polymer creating smaller chains. Cellobiohydrolases (CBH) (also reported as exo-glucanases) cleave these smaller units at the end portion into cellobiose units. β-glucosidases (BG) cleave cellobiose units into individual glucose monomers. For efficient degradation of cellulose, each of these enzymes are required (Kostylev and Wilson, 2012).

Box 1 **Classification of cellulase enzymes.**

Endo- β -1,4-glucanase: Glycosyl hydrolase which has an open-ended catalytic side to randomly bind and cleave the internal β1-4 linkages of cellulose polymer chains producing reduced ends. Commonly called “cellulose.”

Exo- β -1,4-glucanase (aka Cellobiohydrolase): Similar to its Endo- counterpart, except cellulose polymers are threaded through the enzyme to reach the catalytic site.

β-glucosidase (aka cellobiases): Glucosidase enzyme which hydrolyzes the β1-4 linkage cellobiose molecules releasing two glucoses.

If transgenic plants are to be used to overproduce GHs, there are three important aspects to be considered for the host plant: (1) as a biocatalyst production unit, (2) altered cell walls, and (3) auto-hydrolysis of biomass (Taylor et al., 2008; Furtado et al., 2014; Lambertz et al., 2014; Damm et al., 2016). Utilizing plants as enzyme factories is an intriguing concept. It may not be practically feasible in this case, so using plant cell cultures for enzyme overproduction has been proposed (Hellwig et al., 2004). The highest potential goal is to produce bioenergy feedstocks with altered cell walls for high saccharification and their own saccharification enzymes in tandem. An autohydrolytic plant system would require enzymes to be inactive, induced, or sequestered until harvest in order to not negatively affect plant growth. Research on GHs produced in plants is in its relative infancy.

In this review we will highlight successes and challenges of GH production in plants. Here, we compare past use of fungal and bacterial GHs produced in plants and focus on lessons that can inform on selection of appropriate expression systems. We focus especially on the types and properties of insect-derived GHs and assess their potential use in plants. Strategies and recommendations for conducting research and development of CWD enzymes in plants will be discussed with an eye toward improving bioenergy crops and integrated biofuel systems.

## Endo-glucanases in transgenic plants

Heterologous expression of lignocellulosic enzymes *in planta* for improved production of biofuels was proposed in the 1990s, but relatively few enzymes have been tested (Lopez-Casado et al., 2008; Furtado et al., 2014; Lambertz et al., 2014). Mostly, bacterial and fungal enzymes have been overproduced in plants to assess efficacy of the concept (Taylor et al., 2008; Lambertz et al., 2014). Endo-glucanases (EG) have, by far, been the most abundant class of CWD enzymes produced in plants (Table 1). The E1 cellulase from the bacterium *Acidothermus cellulolyticus* has been the most frequent EG produced in transgenic plants (15 papers; Table 1). One reason behind the attention given to E1 is that it is a hyperthermophilic enzyme, which would be inactive during temperatures favoring plant growth.

**Table 1:** **Endo- β -1,4-glucanase transgenic plant studies in which various host plant species and subcellular localization were targeted for enzyme overproduction**.

**Enzyme Name**	**Source organism**	**Host plant**	**Organelle targeting**	**GHF**	**Temp. optimum**	**pH optimum**	**Substrate**	**Activity**	**Notes**	**Reference**
E1	*Acidothermus cellulolyticus*	*Arabidopsis thaliana*	Apoplast	5	65°C n.o.	5.5 n.o.	CMC≠ MUC	26% TSP		Ziegler et al., 2000
E1	*Acidothermus cellulolyticus*	*Lemna minor* 8627	Cytosol	5	80°C	5.0	CMC	0.24 U/g fresh tissue		Sun et al., 2007
E1	*Acidothermus cellulolyticus*	*Nicotiana tabacum*	Chloroplast	5	81°C	5.25	MUC	2756.3 pmol MU mg^−1^ TSP min^−1^		Dai Z. et al., 2000
E1	*Acidothermus cellulolyticus*	*Nicotiana tabacum*	Apoplast	5	81°C	5.25	MUC	18056 pmol MU mg^−1^ TSP min^−1^		Dai et al., 2005
E1	*Acidothermus cellulolyticus*	*Nicotiana tabacum*	Chloroplast	5	65°C n.o.	5.5 n.o.	MUC	2824 FU		Jin et al., 2003
E1	*Acidothermus cellulolyticus*	*Nicotiana tabacum*	Apoplast Chloroplast	5	65°C n.o.	5.5 n.o.	CMC	1.6% TSP	Catalytic domain	Ziegelhoffer et al., 2001
			Cytosol				MUC			
E1	*Acidothermus cellulolyticus*	*Nicotiana tabacum, Zea mays*	Apoplast	5	50°C n.o.	5.0 n.o.	MUC	0.3 ng/mg biomass	Tobacco provided by (Ziegelhoffer) corn provided by Biswall	Brunecky et al., 2011
E1	*Acidothermus cellulolyticus*	*Oryza sativa*	Apoplast	5	65°C n.o.	5.5 n.o.	MUC	25000 pmol MU/mg protein/min		Chou et al., 2011
E1	*Acidothermus cellulolyticus*	*Oryza sativa*	Apoplast	5	65°C n.o.	5.5 n.o.	MUC	4.9% TSP		Oraby et al., 2007
							CMC	0.6 g/L glucose released		
							Avicel	0.25 g/L glucose released		
E1	*Acidothermus cellulolyticus*	*Oryza sativa*	Endosperm	5	80°C	5.0	CMC	830 U/g seed		Zhang Q. et al., 2012
E1	*Acidothermus cellulolyticus*	*Zea mays*	Endosperm cell wall	5	50°C n.o.	5.0 n.o.	MUC	16% TSP	First 40 codons optimized	Hood et al., 2007
E1	*Acidothermus cellulolyticus*	*Helianthus annuus*	Apoplast	5	65°C n.o.	5.5 n.o.	MUC	2.5 mg/kg fresh tissue	Plant codon optimized	Jung et al., 2014
E1	*Acidothermus cellulolyticus*	*Solanum tuberosum*	Chloroplast	5	55°C n.o.	5.5 n.o.	MUC	61000 pmol mg^−1^ min^−1^		Dai Z. Y. et al., 2000
E1	*Acidothermus cellulolyticus*	*Zea mays*	Apoplast	5	65°C n.o.	5.5 n.o.	MUC	0.845 nmol/μg/min	Catalytic domain	Biswas et al., 2006
E1	*Acidothermus cellulolyticus*	*Zea mays*	Apoplast	5	50°C n.o.	4.8 n.o.	MUC	1.13% TSP	Catalytic domain	Ransom et al., 2007
Cel6A	*Thermobifida fusca*	*Medicago trunculata*, *Nicotianatabacum*, *Solanum tuberosum*	Cytosol	6	55°C n.o.	5.5 n.o.	Avicel CMC Corn stover	0.37 g/L 0.47 g/L 0.18 g/L		Ziegelhoffer et al., 1999
							CMC Corn stover	0.47 g/L 0.18 g/L		
EG	Bacteria-like synthetic	*Saccharum officinarum*	Chloroplast ER Vacuole	5	40°C n.o.	4.75 n.o.	CMC	23 nmol glucose/min/mg protein¥		Harrison et al., 2011
ENG1	*Oryza sativa*	*Oryza sativa*	Cytosol	9	n.d.	n.d.	MUC	n.d.	No transgenic plants regenerated	Nigorikawa et al., 2012
t-EGI	*Ruminococcus albus*	*Nicotiana tabacum*	Cytosol	5	30°C n.o.	5.6 n.o.	CMC	1.7 μmol/mg protein	Truncated enzyme	Kawazu et al., 1999
Cel6A	*Thermobifida fusca*	*Nicotiana tabacum*	Chloroplast	6	50°C n.o.	5.5	CMC BMCC	0.65 μm glucose released¥		Yu et al., 2007
sso1354	*Sulfolobus solfataricus*	*Nicotiana tabacum*	Apoplast ER	12	90°C	4.5	CMC≠, Azo-CMC€ MUC Avicel	488 nmol 4MU min^−1^ mg^−1^ 0.1 g/L 5.5 mg L^−1^ h^−1^		Klose et al., 2012
TrCel5A	*Trichoderma reesei*	*Nicotiana tabacum*	Apoplast	5	55°C	4.8	CMC≠	27 nmol 4MU min^−1^ mg^−1^	Ethanol-inducible	Klose et al., 2013
							Azo-CMC€			
							MUC			
TrCel5A	*Trichoderma reesei*	*Nicotiana tabacum*	Apoplast ER	5	55°C n.o.	4.8 n.o.	Azo-CMC	2.1 U mg^−1^ 46 nmol 4MU min^−1^ mg^−1^		Klose et al., 2015
							MUC			

*GHF represents glycosyl hydrolase family as defined by CAZy.org. The temperature and pH optimum are listed. Substrate and other abbreviations used in the table are listed below. ER, endoplasmic reticulum; CMC, carboxymethylcellulose; MUC, 4-methylumbelliferyl-β-D-cellobioside; BMCC, bacterial microcrystalline cellulose; n.o., not optimized, used when reference reported single point activity instead of a range; TSP, total soluble protein; U, one unit is the release of 1 μmol substrate per min; FU, Fluorescence units; ≠82, substrate was used only for qualitative stain for activity; ¥, estimated values from published figure when exact numbers not provided; €, used to measure % relativity to a control within the experiment*.

The first report on E1 production in plants was in tobacco in which E1 was targeted to the chloroplast. E1 was active with an optimal temperature of 81°C and pH of 5.25 (Dai Z. et al., 2000). The cauliflower mosaic virus 35S promoter (*35S*) was used to drive E1 gene expression in *A. thaliana* for apoplast targeting (Ziegler et al., 2000). In this study, E1 was accumulated up to 26% of total soluble protein in primary leaves, which is the highest reported for transgenic plant produced hydrolases (Ziegler et al., 2000; Taylor et al., 2008). Tobacco E1 expressing lines were developed for transport to the chloroplast and found that not all parts of the enzyme would translocate, however the catalytic domain was sufficient to maintain enzyme activity (Jin et al., 2003). In an attempt to optimize E1 expression in tobacco, the hybrid constitutive promoter *Mac* (a combination of the Ti plasmid mannopine synthetase and *35S* promoters) and the *RbcS-3C* (Rubisco small sub-unit) promoter were compared; the *RbcS-3C* was ranked higher in performance. However, the highest total soluble protein was reported as only 0.25%. Apoplast-targeted E1 transgenic potato plants produced 2.6% E1 relative to total soluble protein (Dai Z. Y. et al., 2000). Similar studies in maize, using an enhanced version of the *35S* promoter driving the E1 gene resulted in 2.1% (Biswas et al., 2006) and 1.13% (Ransom et al., 2007) E1 accumulation relative to total soluble protein. Even though none of the above studies reported any negative effects to the host plants, E1 transgenic maize (Ransom et al., 2007), and tobacco cell walls developed differently than their respective non-transgenic parents. In each of these two studies increased saccharification was reported. Wildtype and E1 maize expressing lines were exposed to exogenous application of E1 and showed to be equivalent. The proposed reason for this is due to E1 is active *in vivo* during plant growth causing small nicks within the cellulose polymers. Addition of extra exogenous E1 application of enzyme was ineffectual due to the enzyme already hydrolyzing available cellulose recognition sites (Brunecky et al., 2011). In the same study, Brunecky et al. (2011) examined the transgenic E1 corn and tobacco made by Ziegelhoffer et al., (2001) via histological examination and postulated E1 was having an effect on cell wall growth during development (Ziegelhoffer et al., 2001; Brunecky et al., 2011). The presence of biologically active E1 during cell wall synthesis could play a role in reducing cellulose crystallinity or by creating gaps in the cell wall for increased enzyme accessibility (Brunecky et al., 2011). Aforementioned experiments demonstrate the utility of plant produced endoglucanases as a method for cell wall architecture manipulation.

One goal has been to use transgenic plants as biocatalyst producers in a commodity, such as E1 accumulation in maize seeds. The first 40 codons of E1 were codon-optimized in attempt to increase expression and endow tissue specificity (Hood et al., 2007). E1 in maize seed was further targeted to the cell wall, ER, and the vacuole. In the Hood et al. (2007) study, the ER appeared to be the best subcellular localization target, but none of the localizations led to off-effects in maize. Though active, the E1 enzyme was found to be truncated from a ~70 kDa size down to ~40 kDa. Furthermore, stable enzyme was observed after harvesting and drying (Hood et al., 2007). The researchers introgressed the transgenes into elite germplasm (Hood et al., 2012) for further field experiments (Garda et al., 2015). E1 accumulation increased up to seven-fold in the elite germplasm. Field produced E1 in maize seed appeared to be active, thus illustrating an economically-viable platform to overproduce and store E1 until needed.

In rice, E1 has been studied for production in vegetative biomass apoplasts (Oraby et al., 2007; Chou et al., 2011) and seed endosperm (Zhang Q. et al., 2012). Apoplastic targeting appeared to not affect plant growth (Oraby et al., 2007; Chou et al., 2011), whereas the endosperm production strategy led to dwarfing and early flowering (Zhang Q. et al., 2012). While introduction of E1 led to a suboptimal host plant phenotype, there are multiple variables which need to be addressed. In both cases, E1 was targeted to the apoplast, but used two different constitutive promoters and two different germplasms (Oraby et al., 2007; Chou et al., 2011). Chou et al. (2011) reported higher protein yield at 6.1% over Oraby et al. (2007) 4.9%. When E1 was targeted to the endosperm it was active in dried seeds, but seeds were smaller than wild type seed (Zhang Q. et al., 2012). The researchers speculated that lower seed weight is an effect of the transformation process as transgenic rice expressing non-cellulase proteins have a similar issue (Zhang Q. et al., 2012). No other phenotype was reported in the endosperm-targeted rice. Amongst the three cases, only Zhang Q. et al. (2012) performed an optimization experiment to demonstrate that the E1-produced rice maintained its high thermophilic optimum of 81°C.

E1 was transformed into duckweed (*Lemna minor* 8627), an aquatic plant, for accumulation in the cytosol in which extraction buffers were tested for efficacy. Three extraction buffers, sodium citrate (50 mM pH 4.8), sodium acetate buffer (50 mM pH 5), and HEPES (50 mM pH 8), were used and evaluated for their effects on enzyme yield and activity (Sun et al., 2007). The HEPES buffer extraction yielded more total soluble protein compared to other experimental buffers. When the proteins were extracted with citrate buffer and brief heating at 65°C enzyme activity was increased, although total recovered protein yield was lower than when the HEPES buffer extraction was used (Sun et al., 2007).

Transient expression of E1 was performed in sunflower to study the effects of various promoters: *CaMV35S, CMVar* (cucumber mosaic virus “advanced replicating”), and *TRBO* (tobacco mosaic virus RNA-based overexpression vector). Neither the *CMVar* nor *TRBO* led to E1 production. Utilizing the *35S* promoter, it was shown that addition of methyl jasmonic acid led to a four-fold increase in production of active enzyme (Jung et al., 2014).

*Thermobifida fusca* (formerly named *Thermomonospora fusca*), a thermophilic bacterium, produces Cel6A (formerly E2), an EG that was transformed into alfalfa, tobacco, and potato for cytosolic production. Active Cel6A showed no negative phenotype on whole plants. Cel6A was evaluated for thermostability and was found to be still active from 60–65°C, but became inactive at higher temperatures in the absence of substrate which was comparable to commercially produced Cel6A (Ziegelhoffer et al., 1999). In a subsequent study, Cel6A was targeted to tobacco chloroplasts and no morphological changes were observed in the host plant (Yu et al., 2007). Cel6A production resulted in an improved total soluble protein yield (0.6–4%).

Tobacco cells have been used to express two other EGs from bacteria. The t-EGI from *Ruminococcus albus* was targeted to the cytosol of tobacco cells. The t-EGI was active at 30°C. The t-EGI tobacco plants successfully autohydrolysed their cell walls (Kawazu et al., 1999). The EG SSO1354 from *Sulfolobus solfataricus* was successfully expressed in tobacco apoplasts and endoplasmic reticulum. SSO1354 produced in tobacco had highest activity at 90°C and pH 4.5 and showed no growth differences relative to non-transgenic plants. SSO1354 tobacco was tested with ionic liquid pre-treatment solutions and found to still be active (Klose et al., 2012).

The TrCel5A EG from the fungus *Trichoderma reesei* was produced in tobacco under two construct parameters (Klose et al., 2013). The TrCel5A was active under both construct versions at 55°C and pH 4.8 (Klose et al., 2013). Under the control of the *35S* promoter, TrCel5A produced dwarfed tobacco even with apoplast targeting. However, using an ethanol-inducible, *alcA*, promoter, TrCel5A was produced upon induction and the enzyme was indistinguishable from that in 35S-construct plants (Klose et al., 2013). Similar to the research described above in maize, tobacco progeny produced more TrCel5A than their parents (Klose et al., 2015). In this study, TrCel5A was produced in apoplasts or the ER. In both cases, T2 progeny had wrinkled leaves with spotted necrosis, which would be considered important off-target effects (Klose et al., 2015).

The recent additions of synthetically creating genes by combing databases and preparing sequences *in silico* has been used to generate EG. A proprietary, synthetically-designed EG (psEG) was derived from a bacteria population and introduced into sugarcane. The psEG was targeted to chloroplast, ER, and vacuole with the chloroplast being the highest accumulator near 0.05% total soluble protein. The psEG was driven by a constitutive maize phosphoenolpyruvate carboxylase (*Zm-PEPc)* promoter which expressed psEG in the leaves. Unlike the observation in E1 in maize embryos, no truncation of the psEG protein was detected (Hood et al., 2007; Harrison et al., 2011).

## Cellobiohydrolases in transgenic plants

Heterologous overproduction of cellobiohydrolases (CBHs) has not been explored as much as EG in plants, but CBHs are necessary for complete hydrolysis (Table 2). The first CBH evaluated in plants was Cel6B (formerly named E3), from the bacterium *T. fusca* in the cytosol of alfalfa, tobacco, and potato (Ziegelhoffer et al., 1999). While Cel6B was detectable by western blotting and was not deleterious to plant growth, its activity was negligible and the authors declined further characterization (Ziegelhoffer et al., 1999). Although the *Mac* promoter was used for Cel6B relatively low yield was achieved. The reason for low yield could be the lack of a signal peptide to target to an organelle or the need to codon optimize the proteins, which showed improvement in future CBH studies (Hood et al., 2007). In a later study, Cel6B was targeted to tobacco chloroplasts, and no morphological changes were observed in the host plant (Yu et al., 2007). Cel6B had a total soluble protein yield of 0.6–4%. Enzyme activity was measured using crystalline cellulose as the substrate instead of a soluble cellulose derivative substrate (i.e., CMC or MUC) which could explain the low activity reported.

**Table 2:** **Cellobiohydrolase transgenic plant studies in which various host plant species and subcellular localization was targeted for enzyme overproduction**.

**Enzyme name**	**Source organism**	**Host plant**	**Subcellular localization**	**GHF**	**Temp**	**pH**	**Substrate**	**Activity**	**Notes**	**Reference**
CBH I	*Trichoderma reesei*	*Zea mays*	Cell wall, ER, vacuole	7	50°C	5.0	MUC	16% TSP	First 40 codons optimized	Hood et al., 2007
CBH I	Fungal-like synthetic	*Saccharum officinarum*	Chloroplast, ER, vacuole	7	40°C	4.75	MUL	12.25 nmol 4-Mu /min/mg protein		Harrison et al., 2011
CBH II	Fungal-like synthetic	*Saccharum officinarum*	ChloroplastER, vacuole	6	40°C	4.75	Avicel PH-101	8.68 nmol reducing sugars/min/mg protein		Harrison et al., 2011
Cel6A	*Thermobifida fusca*	*Medicago trunculata, Nicotiana tabacum, Solanum tuberosum*	Cytosol	6	55°C	5.5	CMC	0.1% TSP		Ziegelhoffer et al., 1999
Cel6B	*Thermobifida fusca*	*Medicago trunculata, Nicotiana tabacum, Solanum tuberosum*	Cytosol	6	55°C	5.5	CMC	0.02% TSP		Ziegelhoffer et al., 1999
Cel6B	*Thermobifida fusca*	*Nicotiana tabacum*	Chloroplast	6	50°C	5.5	BMCC	0.55 μm glucose released¥		Yu et al., 2007
EXG1	*Oryza sativa*	*Oryza sativa*	Native target	6	37°C	7.0	MUC	45 μg/ml 4-MU concentration		Nigorikawa et al., 2012

*GHF represents glycosyl hydrolase family as defined by CAZy.org. The tested temperature and pH are listed. Substrate abbreviations are listed below. CMC, carboxymethylcellulose; BMCC, bacterial microcrystalline cellulose; MUL, 4-methylumbelliferyl-β -D-lactopyranoside; MUC, 4-methylumbelliferyl-β-D-cellobioside; TSP, total soluble protein; ¥, estimated values from published figure when exact numbers not provided*.

Initial experiments on CBH1 from the fungus *T. reesei* driven by the constitutive *35S* promoter, yielded 0.11–0.82% total soluble protein from tobacco leaves and callus and showed no deleterious effect on plant growth (Dai et al., 1999). Further experiments with CBH1 used the maize embryo promoter, globulin-1, and targeted to the cell walls, ER, and vacuoles for increased accumulation (Hood et al., 2007). The first 40 codons were optimized for improved maize protein synthesis. The combination of tissue, organelle, and codon optimization resulted in up to 16% total soluble protein, which is the highest yield reported for CBH1 (Hood et al., 2007). An issue noted while lines for CBH1 targeted to the ER had enzyme activity, they failed to be present on Western blots. However, the cell wall targeted CBH1 was a full length variant. Further evaluation demonstrated the CBH1 was truncated. In future studies, it may be necessary to fully codon optimize GHs to reduce the risk of truncation, especially in cases where the catalytic domain is near the C-terminus of the protein. Field experiments of maize progeny carrying CBH I, showed no yield or growth performance difference compared to wild type counterparts under field conditions (Garda et al., 2015).

Proprietary synthetic CBH1 and CBHII (psCBH1 and psCBHII) genes were generated using multiple bacterial and fungal sources as templates and were transformed into sugar cane (Harrison et al., 2011). Each protein was targeted to either chloroplast, ER, or vacuole with none reporting any morphological defects. The highest activity was measured from senesced leaves of psCBH1 targeted to the vacuole (Harrison et al., 2011). Both proteins were successfully localized to the leaves by the *Zm-PEPc* promoter and neither of them were truncated (Harrison et al., 2011).

In another study, recombinant synthesis of an endogenous rice CBH (EXG1) resulted in increased glucose release in rice shoots (Nigorikawa et al., 2012). The EXG1 construct utilized its native signal peptide. Some transgenic events were not observably morphologically different than the non-transgenic parent, but three events had deformed, split leaves and extra lacunae. The authors hypothesized that the altered leaf phenotype was from weakened cell walls imbued by the EXG1. Of the 28 EXG1 rice events recovered, 12 were completely sterile, and 14 were partially sterile (defined as < 40 seeds produced; Nigorikawa et al., 2012).

## β-glucosidases in transgenic plants

β-glucosidases (BGs) are necessary for degradation of cellobiose into glucose, and various BG genes have been overexpressed in plants (Table 3). Heterologous expression of the *T. maritime* BglB enzyme resulted in no altered plant morphology in tobacco or Arabidopsis, and the recombinant protein had the characteristic thermophilic properties (Jung et al., 2010, 2013). Crude extract from BglB tobacco was applied to transgenic rice expressing Cel5A endoglucanase, which resulted in improved saccharification in an expected synergistic fashion (Jung et al., 2010). Over-expression of the endogenous BEG1 rice BG in transgenic rice also showed no morphological effects, however no enzyme activity was reported (Nigorikawa et al., 2012). The lack of morphological effects on host plants were hypothesized to be the result of predominant cellobiose degradation and not complete cellulose chains (Jung et al., 2010; Nigorikawa et al., 2012). BGs are the least reported on of the essential CWDs and more investigation into their functional role for a complete autohydrolysis system will be required.

**Table 3:** **β-glucosidase (BG) transgenic plant studies in which various host plant species and subcellular localization was targeted for enzyme overproduction**.

**Enzyme name**	**Source organism**	**Host plant**	**Organelle targeting**	**GHF**	**Temp**.	**pH**	**Substrate**	**Activity**	**Notes**	**Reference**
BEG1	*Oryza sativa*	*Oryza sativa*	Native	1	n.d.	n.d.	n.d.	n.d.		Nigorikawa et al., 2012
BglB	*Thermotoga maritima*	*Nicotiana tabacum* and *Arabidopsis thaliana*	Chloroplast Cytosol	3	40-90°C (80°C)	3-6 (4.5)	*p*NPG	180 mmol/ml¥		Jung et al., 2010
BglB	*Thermotoga maritima*	*Nicotiana tabacum* and *Arabidopsis thaliana*	Chloroplast	3	80°C	4.5	*p*NPG	2090.9 mg/ml	New chloroplast promoter	Jung et al., 2013

*GHF represents glycosyl hydrolase family as defined by CAZy.org. The temperatures and pH ranges tested and (optimum) are listed. Substrate abbreviations are listed below. pNPG, p-nitrophenyl β-D-glucopyranoside; ¥, estimated values from published figure when exact numbers not provided*.

## Bacterial and fungal xylanases in transgenic plants

Xylans are the major carbohydrates in the hemicellulose portion of the plant cell wall. Xylanases break down the bonds of β-1,4-xylan, resulting in simpler pentose sugars that can be fermented to produce biofuels. Dicots and non-commelinoid monocots have Type I cell walls predominately made up of xyloglucan, while grasses are made up of Type II cell walls that contain high levels of arabinoxylan (Scheller and Ulvskov, 2010). Because of the inherent differences, xylanase efficacy for the different cell wall types in the host organism should be considered.

Several plants have been the hosts to heterologously-produced xylanases in plants, including Arabidopsis, barley, potato, rice, sunflower, and tobacco. In these cases the enzymes have maintained their thermophilic and mild acidic to neutral pH properties (Table 4). Overexpression of xylanases have not yielded any plants with observable morphological differences compared to controls.

**Table 4:** **Endo-1,4-β –xylanase transgenic plant studies in which various host plant species and subcellular localization was targeted for enzyme overproduction**.

**Enzyme name**	**Source organism**	**Host plant**	**Organelle targeting**	**GHF**	**Temp**.	**pH**	**Substrate**	**Activity**	**Notes**	**Reference**
ATX	*Thermobifida fusca*	*Oryza sativa*	Cytosol	11	50°C	5.0	Birchwood xylan	3.64 U g^−1^ fresh tissue		Weng et al., 2013
XynA	*Clostridium thermocellum*	*Oryza sativa*	Cytoplasm	11	60°C	7.0	Birchwood xylan	0.475 Unit/mg protein¥	Catalytic domain without native signal peptide	Kimura et al., 2003
XynA	*Dictyoglomus thermophilum*	*Arabidopsis thaliana, Nicotiana tabacum*	Apoplast	11	85°C	6.5	Wheat arabinoxylan	8.25 μmol reducing ends/min/mg¥	Plant codon optimized	Borkhardt et al., 2010
XynA	*Dictyoglomus thermophilum*	*Arabidopsis thaliana, Nicotiana tabacum*	Apoplast	11	85°C	6.5	Wheat arabinoxylan	8.25 μmol reducing ends/min/mg¥	Plant codon optimized	Borkhardt et al., 2010
mXynA	*Neocallimastix patriciarum*	*Hordeum vulgare*	Cytoplasm	11	40°C	6.0	AZCL-xylan	27.06 AU/h/grain		Patel et al., 2000
XylII	*Trichoderma reesei*	*Arabidopsis thaliana*	Peroxisome, Cytosol, Chloroplast	11	50°C	6.8	Xylan solution	24 U mg^−1^		Hyunjong et al., 2006
XynII	*Trichoderma reesei*	*Arabidopsis thaliana*	Cytosol, Chloroplast	11	50°C	6.8	Xylan solution	22.7 U/mg		Bae et al., 2008
XynB	*Streptomyces olivaceoviridis*	*Solanum tuberosum*	Cytosol	11	60°C	5.2	Birchwood xylan	87 μmol min^−1^ g-1		Yang et al., 2007
XynZ	*Clostridium thermocellum*	*Nicotiana tabacum*	Apoplast	10	60°C	5.4	RBB-Xylan, Birchwood xylan, Oat spelt	46.6 U/mg	Truncated	Herbers et al., 1995

*GHF represents glycosyl hydrolase family as defined by CAZy.org. The temperature and pH are values enzyme activity was performed. Substrate and other abbreviations are listed below. AZCL-xylan, azo-crosslinked-xylan; RBB-xylan, remazol brilliant blue R-D-xylan; U, amount of enzyme liberating 1 μmol xylose per min; AU, absorbance unit; ¥, estimated values from published figure when exact numbers not provided*.

A codon-optimized *xynA* gene from the fungus *Neocallimastix patriciarum* was expressed in barley under the control of two separate endosperm-specific promoters (*GluB-1* & *Hor2-4*) (Patel et al., 2000). The *GluB-1* version had twice the expression and activity of the other version in mature seed, with no expression observed in leaf, stem, or root tissues. Dried and stored seed maintained xylanase activity, which would enable biofuel catalyst production or direct feeding to animals. Two *Clostridium thermocellum* xylanases have been produced in transgenic plants. The first was a truncated xylanase (XynZ) produced in tobacco apoplasts, in which thermostability was maintained (Herbers et al., 1995). XynZ was active against multiple xylan substrates at high temperature and slightly acidic pH. The second was XynA produced in rice, which accumulated in the cytoplasm. The native signal peptide was removed, but the catalytic domain was left intact. The recombinant enzyme had native-like activity at neutral pH and thermophilic temperature of 60°C (Kimura et al., 2003). In these experiments, the enzyme accumulated in seed was active after dry storage. In Arabidopsis, two different xylanases from the fungus *T. reesei* were used to overproduce high levels of enzyme. The first, XYLII, accumulated in the cytosol, peroxisome, and chloroplast, depending on the experiment. Co-localization into chloroplast and peroxisomes resulted in the highest yield of XYLII (Hyunjong et al., 2006). The second xylanase tested in Arabidopsis was XYNII, which was targeted to either the chloroplast or the cytosol. Chloroplast-targeted XYNII yielded the higher amounts of protein compared with the non-targeted variant. Plant-derived XYNII was found to be comparable to a commercial xylanase in activity under lab conditions (Bae et al., 2008). Finally, *Streptomyces olivaceoviridis* XynB was overproduced in potato cytosol or apoplast. XynB was stable at 60 and 70°C and subsequent generations of the transgenic potato produced greater amounts of XynB than the T0 generation (Yang et al., 2007).

## Combining glycosyl hydrolase genes in transgenic plants for improved conversion performance

Cellulolytic enzymes have been combined to assess potential synergistic effects by gene stacking in transgenic plants (Table 5). Tobacco has been the most often-used host plant, but the first reported host for stacked GHs was barley (Jensen et al., 1996; Fan and Yuan, 2010; Mahadevan et al., 2011; Lee et al., 2012). The first experiments in barley were accomplished using protein fusions. The EII-hybrid construct is a plant codon-optimized fusion of two bacterial enzymes from *Bacillus spp*. glucanase and a *Bacillus spp*. α-amylase for expression in barley. EII-hybrid was expressed in endosperm during germination under the control of the endogenous (barley) EII promoter and the the high-pI-α-amylase signal peptide (Jensen et al., 1996). In another study, the same EII-hybrid gene construct was driven by *Hor3-1* endosperm promoter with the Hor3-1 native signal peptide (Horvath et al., 2000). Neither EII-hybrid construct seemed to cause any morphological changes to plant growth (Jensen et al., 1996; Horvath et al., 2000). EII-hybrid was active at pH 7.4 and 65°C (Horvath et al., 2000; Jensen et al., 1996). Another barley transgenic hydrolase named *cel-hyb1* is a fusion of an EG from the fungus *N. patriciarum* and a 1,3-1,4-β-glucanase from *Piromyces sp*. (Xue et al., 2003). The Cel-hyb1 transgenic lines showed no observable morphological defects to plant health while maintaining equal enzyme activity for fresh or dried biomass. Cel-hyb1 had a lower temperature activity at 40°C and slightly more acidic pH (Xue et al., 2003). Both cases demonstrated the use of barley as a functional bioreactor and could be used for production of transgenic cellulases or improved feedstocks for animals (Xue et al., 2003).

**Table 5:** **Transgenic plants producing gene stacked cellulolytic enzymes produced in plants as reported in the literature**.

**Enzyme name**	**Source organisms**	**Host plant**	**GHF**	**Temp. (°C)**	**pH**	**Substrate**	**Organelle targeting**	**Notes**	**Reference**
EII-hybrid	*Bacillus* spp.	*Hordeum vulgare*	16	65	7.4	Azobarley β-glucan (40 ng ± 18 ng) Lichenan≠,	Endosperm	Codon optimized	Jensen et al., 1996
EII-hybrid	*Bacillus* spp.	*Hordeum vulgare*	16	65	7.4	Azobarley β-glucan (Up to 40 μg^*^mg^−1^) Lichenan≠,	Endosperm	Codon optimized	Horvath et al., 2000
E1 & Xyn10A	*Acidothermus cellulolyticus*	*Helianthus annuus*	5 & 10	70	6.0	MUC (up to 2.5 mg/kg)	Apoplast	Plant codon optimized	Jung et al., 2014
						Birchwood xylan (up to 4.1 mg/kg)¥			
BglB:Cel5A	*Thermotoga maritime*	*Nicotiana tabacum*	3 & 5	80	5.0	CMC (0.375 mg/ml) ¥	Chloroplast		Lee et al., 2012
						*p*NPG (240 μmol/ml) ¥			
XylII:Cel5A	*Trichoderma reesei* and *Thermotoga maritime*	*Nicotiana tabacum*	11 & 5	50	7.0	CMC (0.275 mg/ml) ¥	Chloroplast		Lee et al., 2012
						Oat spelt (0.25 mg/ml) ¥			
Cel6B:Cel5A	*Thermobifida fusca* and *Thermotoga maritime*	*Nicotiana tabacum*	5 & 6	50	7.0	CMC (0.375 mg/ml) ¥	Chloroplast		Lee et al., 2012
						Filter paper (0.225 mg/ml) ¥			
Cel-Hyb1 (CelA & Cel6G)	*Neocallimastix patriciarum* and *Piromyces* sp.	*Hordeum vulgare*	& 6	40	6.0	CMC≠,	Cytoplasm		Xue et al., 2003
						AZCL-glucan (8447 ± 2059 AU/h/g/ grain)			
						AZCL-HE-Cellulose (4041 ± 657 AU/h/g/ grain)			
						Lichenan (1585 ± 195 U/g grain			
TmCel5A & TmCel5Afused CMB6A	*Thermotoga maritime*	*Nicotiana tabacum*	5 & CBM6	50	6.0	CMC (up to 2.0 Unit/mg tsp)¥	Apoplast, Chloroplast, Cytosol	Fused with cellulose binding module	Mahadevan et al., 2011
Xyln-ara	*Clostridium thermocellum* and *Geobacillus stearothermophilus*	*Nicotiana tabacum*	10 & 51	60	6.0	RBB-xylan (55.3 μmol dye/min/μg) Wheat-AX (22.5 μmol xylose/min/μg) ¥Rye-AX (27.5 μmol xylose/min/μg)¥	Cytosol	Fusion of catalytic domains	Fan and Yuan, 2010

*Enzyme name is the name given by the author, source organisms are where the enzymes originated from, host plant is the plant species which was transformed with the enzyme, GHF is the glycosyl hydrolase family number as defined by CAZy.org, temperature, and pH are values enzyme activity was performed, substrate is the material used to measure enzyme activity followed by parenthesis of reported highest mean activity, organelle targeting is where enzyme was targeted toward in the plant cell, activity is the reported maximum activity provided by authors, notes list other key interests for the reference, reference is the referred paper information was collected*.

*CBM, carbohydrate binding module; CMC, carboxymethylcellulose; MUC, methylumbelliferyl-β-D-cellobioside; pNPG, p-nitrophenol; RBB-xylan, Remazol brilliant blue; AX, arabinoxoylan; AZCL = azo-crosslinked; tsp, total soluble protein; AU, absorbance unit; ≠82 substrate was used only for qualitative stain for activity; ¥, estimated values from published figure when exact numbers not provided*.

A hybridized xylanase-arabinase construct (Xyln-ara) was developed by fusing the catalytic domains of a xylanase from *C. thermocellum* and an arabinofuranosidase from *Geobacillus stearothermophilus* and introduced into tobacco. The fusion of the domains revealed novel enzyme character for having an improved activity at pH 6-9 when compared to the original enzyme. The fused enzymes demonstrated activity on carboxymethyl cellulose (CMC) substrate while the native forms did not. The Xyln-ara hybrid was targeted to the cytosol and showed no effect on plant growth (Fan and Yuan, 2010). Use of catalytic domains is advantageous by reducing the size of transformation constructs which improves multi-gene integration experiments and down-stream analysis (Fan and Yuan, 2010).

From *T. maritima*, the Cel5A endoglucanase and an engineered CBM6-Cel5A fusion hydrolase construct were introduced and compared in transgenic tobacco. CBM6 is a carbohydrate binding module from CBM family 6 of xylanase A in *C. stercorarium*. When the CBM6-Cel5A was first engineered and tested in *E. coli* it showed up to an 18-fold increase in activity over non-fused Cel5A and was selected for tobacco evaluation (Mahadevan et al., 2008). The CBM6-Cel5A was targeted to the chloroplast, apoplast, and cytosol where the chloroplast targeted enzyme had the highest accumulation and no morphological defect was reported (Mahadevan et al., 2011). Autohydrolysis of plant material was performed on biomass and found the enzymes were able to have increased release of glucose in plants with the hybrid and native gene (Mahadevan et al., 2011).

In tobacco, BglB, Cel5A, XylII, or Cel6B were connected by the linker from the 2A oligopeptide from the foot-and-mouth disease virus to test a gene stack integration to improve hydrolysis. It was noted that selected hydrolase genes did have a pH dependent effect on hydrolysis. Combination of these enzymes worked well when on CMC substrate, however they showed low activity on filter paper (Lee et al., 2012). A synergistic effect was observed when BglB:Cel5A and Cel6B:Cel5A were co-expressed, but not observed for XylII:Cel5A.

In sunflower E1 and the xylanase Xyn10A from *A. cellulolyticus* were transiently co-expressed to the leaf apoplast using agroinfiltration for potential applications of biofuel production (Jung et al., 2014). Transient expression of both enzymes was achieved using the *CaMV35S* constitutive promoter, however yields were very low compared to previously reported plant expression systems (Taylor et al., 2008; Jung et al., 2014). Equivalent agroinfiltration experiments using *CMVar* and *TRBO* promoters for both enzymes were failures, but expression was achieved in this system upon methyl jasmonic acid treatment and increasing temperature from 20 to 30°C (Jung et al., 2014).

Future work on gene stacking GHs in plants should combine each enzyme grouping to constitute a complete autohydrolysis system. Selected GHs would need plant codon optimization for improved yield and prevent truncation (Hood et al., 2007). Organelle targeting to either the apoplast or the vacuole would be the best for high protein accumulation (Ziegler et al., 2000; Hood et al., 2007). Use of precision genome editing tools could knock out non-essential growth pathways to increase protein yield (Liu et al., 2014). For example, E1 targeted to maize endosperm cell walls could be mutated to reduce starch storage in favor of storing E1. Adding in CBHs and BGs creates the enzymatic synergy needed for complete autohydrolysis.

## Insect bioprospecting avenues for transgenic plant produced hydrolases

Insects are a relatively recent source for bioprospecting biocatalysts for biofuel production (Carroll and Somerville, 2009; Oppert et al., 2010). Isopteran (termite) digestive tracts are very efficient: 99% of ingested cellulose and 87% of hemicellulose is converted into usable sugars (Ohkuma, 2003). Researchers once believed that insects produced few-to-no endogenous CWD enzymes, but rather relied on those from symbiotic organisms in their guts to break down plant biomass. For some species this is still believed to be the case, however genomic and proteomic analyses have shown that insects produce endogenous enzymes (Watanabe et al., 1998; Tokuda and Watanabe, 2007). For example, termites can survive solely on cellulose, and thus would be a source of cell wall digesting enzymes for bioprospecting (Watanabe and Tokuda, 2010). Heterologous expression of insect cellulases in model organisms have shown them to be potential alternatives to bacterial and fungal-sourced enzymes. Many insect cellulases reported in the literature have temperature optima from 40 to 65°C and perform optimally at alkaline pH (Shi et al., 2011, 2013; Willis et al., 2011). High temperature and variable pH characteristics were discovered as researchers compared insect derived hydrolases to microbial hydrolases. These high temperature and variable pH could be from the native structure formed by GHs or from the symbiont ancestors which resided in the insect digestive system.

Insect cellulolytic enzymes fall into the GH families 1, 5, 9, 11, and 45. The majority of insect cellulases discovered are from the Coleoptera (beetle) and Isoptera (termite) taxonomic orders as defined by Misof et al. (2014). In addition, Blattodea (roaches) Phasmatodea (stick insects), Lepidoptera (butterflies/moths), and Orthoptera (grasshopper/crickets) have demonstrated cellulolytic activity (Oppert et al., 2010; Shelomi et al., 2014). Endogenous and insect endosymbiont derived enzymes have similar characteristics to bacterial and fungal derived enzymes in terms of thermostability and variable pH (Oppert et al., 2010). Insect derived enzymes often have a more alkaline pH range than those from microbial sources, since the insect digestive systems can be up to a pH of 12 in the insect digestive systems (Dow, 1992; Brune, 2014).

The digestive systems of insects are broken into three distinct sections of foregut, midgut, and hindgut. The foregut includes the mouth, salivary glands, and the most anterior chamber of the digestive tract. Cellulolytic enzymes have been discovered in the salivary glands which along with the maceration from the mandibles begins initial degradation. The fore- and midgut contain mostly endogenous cellulases (Slaytor, 1992; Brune, 2014). Malpighian tubules, when present, separate the mid and hindgut. The majority of microbial symbionts have been discovered in the hindgut. Specifically, protist and bacterial cellulases have been isolated from the hindgut of Coleoptera, Isoptera, and Orthoptera insect orders (Watanabe et al., 1998; Brune, 2014). Both symbiont and endogenous insect GH systems have the potential for use as novel biocatalysts.

A major impediment to identifying and cloning insect CWD enzyme-coding genes is the lack of sequenced and assembled genomes among insect species, especially those that have a diet consisting of plant cell walls. *Apis melliferia, Drosophila melanogaster, Drosophila pseudoobscura, Tribolium castaneum, Nasonia* spp., *Acyrthosiphon pisum*, and *Heliconius* spp. are taxa with the most complete genome data (Richards et al., 2005; Weinstock et al., 2006; Tribolium Genome Sequencing et al., 2008; International Aphid Genomics Consortium, 2010; Werren et al., 2010; Heliconius Genome Consortium, 2012). Of these, only *T. castaneum* and *A. pisum* and, *Heliconius* spp., are herbivores. There are efforts, Such as the “i5K” to sequence and assemble 5000 insect genomes, of which 28 pilot studies are underway and new phylogenetic trees are being designed (Robinson et al., 2011; Misof et al., 2014; Behura, 2015). To advance insect CWD gene discovery, herbivorous insects and their metagenomes should be sequenced to advance the field beyond its present state (Table 6).

**Table 6:** **Heterologously produced insect cellulolytic enzymes as reported in the literature**.

**Order: family**	**Genus species**	**Enzyme (family)**	**Temp. (°C)**	**pH**	**Substrate**	**Activity**	**Expression system**	**Reference**
**COLEOPTERA**								
Cerambycidae	*Apriona germari*	EG (GH 45)	50	6.0	CMC	992 (U/mg)	Sf9 insect cells	Lee et al., 2004
Cerambycidae	*Apriona germari*	EG (GH 45)	50	6.0	CMC	812 (U/mg)	Sf9 insect cells	Lee et al., 2005
Cerambycidae	*Apriona germari*	EG (GH 5)	55	6.0	CMC	1037 (U/mg)	Sf9 insect cells	Wei et al., 2006
Cerambycidae	*Batocera horsfieldi*	EG (GH 45)	50	6.0	CMC	928 (U/mg)	baculovirus expression in *Bombyx mori*	Xia et al., 2013
Cerambycidae	*Anoplophora malasiaca*	EG (GH 45)	50	4.0	CMC	319.22 ± 9.3 (U/mg)	baculovirus expression in *Bombyx mori*	Chang et al., 2012
Chrysomelidae	*Diabrotica virgifera virgifera*	EG (GH 45)	45	6.0	CMC	166.67 nmol min^−1^ mgprotein^−1^	*E. coli*	Valencia et al., 2013
Tenebrionidae	*Tribolium castaneum*	EG (GH 9)	50	9.0	CMC	12.9 (U/mg)	S2 insect cells	Willis et al., 2011
**ISOPTERA**								
Kalotermitidae	*Neotermes koshunensis*	BG (GH1)	45	5.0	Cellobiose	156.7 (U/mg)	*E. coli*	Ni et al., 2007; Uchima et al., 2011
			40	5.0	pNPG	12.4 (U/mg)	*A.orizae*	
Rhinotermitidae	*Coptotermes formosanus*	EG (GH5)	70	6.0	CMC	105 (U/mg)	*E. coli*	Inoue et al., 2005
Rhinotermitidae	*Coptotermes formosanus*	EG (GH9)	43	5.6	CMC, Cellodextrin, Filter paper	325 (U/mg)	*E. coli*	Zhang D. et al., 2011
Rhinotermitidae	*Coptotermes formosanus*	BG (GH1)	49	5.6-6.2	Cellobiose	462.6 (U/mg)	*E. coli*	Zhang D. et al., 2012
Rhinotermitidae	*Reticulitermes flavipes*	EG (GH9)	50–60	6.5-7.5	CMC	1.3 (U/mg)	baculovirus expression in *Trichoplusia ni*	Zhou et al., 2010
Rhinotermitidae	*Reticulitermes flavipes*	BG (GH1)	40	6.5-7.0	Cellobiose	638.0 ± 39.0 (U/mg)	baculovirus expression in *Trichoplusia ni*	Scharf et al., 2010
Rhinotermitidae	*Reticulitermes santonensis*	BG (GH1)	40	6.0	pNPG	0.441 (U/mg)	*E.coli*	Mattéotti et al., 2011
Rhinotermitidae	*Reticulitermes santonensis*	Xyl (GH11)	55	5.0	AZCL-xylan, Beechwood xylan	1837 (U/mg)	*E. coli*	Mattéotti et al., 2012
Rhinotermitidae	*Reticulitermes speratus*	EG (GH9)	45	5.5	CMC	1200 ± 92 (U/mg)	*A.oryzae*	Hirayama et al., 2010
Rhinotermitidae	*Reticulitermes speratus*	EG (GH7)	45	6.5	CMC	603 (U/mg)	*A. oryzae*	Todaka et al., 2010
Termitidae	*Globitermes sulphureus*	BG (GH1)	90	6.0	pNPG	110 (U/mg)	*E.coli*	Wang et al., 2012
Termitidae	*Macrotermes annandalei*	Xyl (GH11)	55	7.5	Beechwood xylan	733 (U/mg)	*E.coli*	Liu et al., 2011
Termitidae	*Macrotermes barneyi*	BG (GH1)	45	5.0	Cellobiose, pNPG	206 (U/mg)	*E.coli*	Wu et al., 2012
Termitidae	*Nasutitermes takasagoensis*	EG (GH9)	65	6.0	CMC	1392 ± 57 (U/mg)	*A.orizae*	Hirayama et al., 2010
Termitidae	*Nasutitermes takasagoensis*	BG (GH1)	65	5.5	pNPG	5.83 (U/mg)	*P. pastris*	Uchima et al., 2012
**LEPIDOPTERA**								
Noctuidae	*Spodoptera frugiperda*	BG (GH1)	30	6.0	*p*NPG	2.4 (mM^−1^s^−1^)	*E. coli*	Marana et al., 2004
**ORTHOPTERA**								
Acrididae	Grasshopper (n.r)	BG (GH 1)	60	9.0	*p*NPG	2.61 ± 0.75 (mM^−1^s^−1^)	*E. coli*	Shi et al., 2011
Gryllidae	*Teleogryllus emma*	EG (GH9)	40	5.0	CMC	3118.4 (U/mg)	Sf9 cells	Kim et al., 2008

*Insect taxanomic order followed by insect family. Genus species are insect source of enzyme, Enzyme and family represents glycosyl hydrolase and its corresponding family as defined by CAZy.org, temperature and pH are values reported for best performance, substrate is the material used to measure enzyme activity, heterologous expression system defines transformation system evaluated, reference is the referred paper information was collected*.

*EG, endo-1,4-β-glucanase; BG, β-glucosidase; Xyl, Xylanase; U, units of mMol of substrate released per minute; n.d., not determined; n.r., not reported; n.o., not optimized, used when references reports activity, however was a single temperature or pH testing*.

Termites are the archetypical wood-consuming insects. The first endogenous insect cellulase was discovered from the genome of the termite, *Reticulitermes speratus* (Watanabe et al., 1998). Since then many other termite-derived hydrolases have been discovered and even evaluated for heterologous expression. Isoptera families are split into the lower termites (Mastotermitidae, Kalotermitidae, Hodotermitidae, Termopsidae, Rhinotermitidae, and Serritermitidae) and higher termites (Termitidae). The distinction between these families is based on presence of flagellated protist symbionts in the hindgut (lower termites) or their absence (higher termites) (Ni and Tokuda, 2013; Brune, 2014). The “lower vs higher” termite digestion distinction is the basis of evolutionary importance being that the higher termites evolved later (Slaytor, 1992; Watanabe and Tokuda, 2001; Ni and Tokuda, 2013). A prevailing theory on this change is due to ecological changes which caused Isoptera ancestors' loss of protists symbionts, change of habitat, and/or food source and possible reacquisition of different cellulase-harboring symbionts (Ni and Tokuda, 2013; Slaytor, 1992; Watanabe and Tokuda, 2001). In either case of both higher and lower termites have provided cellulolytic enzymes which could be utilized for autohydrolysis plants.

GHs isolated from two of the lower termite families, Kalotermitidae and Rhinotermitidiae, have been evaluated in heterologous systems. From Kalotermitide, a *Neotermes koshunensis* BG (NkBG) was produced in *E. coli* and in *Aspergillus oryzae*. NkBG was functional in both systems, however it had a higher activity from *E. coli* (156.7 U/mg) compared to *A. oryzae* (12.4 U/mg). NkBG produced in *A. oryzae* had a unique property for maintaining 100% activity in the presence of 0.6 M glucose; most BG enzymes are inhibited by excess glucose (Uchima et al., 2011). Rhinotermitidae enzymes have been heterologously expressed from *Coptotermes formosanus, Reticulitermes flavipes, Reticulitermes santonensis*, and *R. speratus*. From *C. formosanus* two EGs (CFP-EG1and CfEG5) were evaluated in *E. coli*. CFP-EG1 is from a protist symbiont and had a thermophilic activity at 70°C with a CMC substrate activity of 105 U/mg (Inoue et al., 2005). Conversely, CfEG5 had three-fold higher CMC activity at 325 U/mg, but was less thermally active with an optimum of 43°C (Zhang D. et al., 2011). A BG from *C. formosanus* was expressed in *E. coli* and demonstrated an activity of 462.6 U/mg on cellobiose (Zhang D. et al., 2012). From *R. flavipes*, an EG (Cell-1) and a BG (RfBGluc-1) were expressed using the baculovirus expression system in which *Trichoplusia ni* larvae served as bioreactors. Hemolymph from baculovirus-injected larvae were extracted and analyzed for enzyme activity (Scharf et al., 2010). Cell-1 CMC activity of 1.4 U/mg was low while RfBGluc-1 was considerably more effective on cellobiose at 638 U/mg. An opposite trend was seen from an *R. santonensis* BG which had a low activity of 0.441 U/mg against *p*-nitrophenol-β-D-glucopyranoside (pNPG) subtstrate (Mattéotti et al., 2011). Possibly this was due to being produced in *E. coli* instead of *T. ni*. Also from *R. santonensis* is the xylanase, mXylB8, which has the highest reported beechwood xylan activity at 1837 U/mg for an insect derived xylanase with a mildly acidic range of pH 5.0 and temperature of 55°C (Mattéotti et al., 2012). From *R. speratus* two EGs (RsEG and RsSymEG1) were produced in *A. oryzae* both of which were active at 45°C and a CMC activity of 1200 and 605 U/mg, respectively (Hirayama et al., 2010; Todaka et al., 2010). RsEG is an endogenous cellulase and RsSymEG1 is from a protist symbiont. It is interesting to note that the endogenous RsEG is roughly double the activity of the symbiont.

The higher termite family, Termitidae, has also provided useable GHs for plant biocatalysts engineering. The BG, bg1-gs1 from *Globitermes sulphureus*, was produced in *E. coli* and demonstrated a thermophilic activity at 90°C producing 110 U/mg from pNPG substrate (Wang et al., 2012). While not having as high a thermal stability, the MbmgBG1 from *Macrotermes barneyi*, had a higher activity of 206 U/mg on cellobiose with a more acidic pH of 5.0 at temperature of 45°C (Wu et al., 2012). Another thermophilic insect derived BG is the G1mgNTBG from *Nasutitermes takasagoensis*, while optimum temperature is 65°C, the activity was the lowest, 5.83 U/mg, when produced by *Pichia pastoris* (Uchima et al., 2012). Also from *N. takasagoensis*, is the EG, NtEG, which when produced in *A. oryzae* produced the highest CMC activity at 1392 U/mg at a slightly acidic pH of 6.0 at 65°C (Hirayama et al., 2010). A xylanase from *Macrotermes annandalei*, was produced in *E. coli* and evaluated by digestion of beechwood xylan to produce 733 U/mg at a near neutral 7.5 pH and 55°C (Liu et al., 2011). Both higher and lower Isopteran species have proven to be a reliable source of GHs, which should be evaluated for their efficacy in plant based expression systems.

Coleopteran derived GHs are found predominantly in the larval stages. The wood-boring *Apriona germari* has provided three different EGs all with heterologous activity from Sf9 insect cells. When using CMC as the substrate, AgEG-I, AgEG-II, and AgEG-III produced activities of 992, 812, 1037 U/mg, respectively (Lee et al., 2004, 2005; Wei et al., 2006). A baculovirus transformation system into *Bombyx mori* larvae was used to heterologously express two other wood-boring beetle cellulases. Hemolymph extracted from transformed larvae carried the EGs when tested on CMC produced activity levels of 927 U/mg for *Batocera horsfieldi* EG and 319.22 ± 9.3 U/mg for *Anoplophora malasiaca* EG (Chang et al., 2012; Xia et al., 2013). Larvae from *Diabrotica virgifera virgifera* were examined as a biocatalyst source due to their diet of maize stems which could be used as a biofuel source. An EG from *D. virgifera* was isolated and produced in *E. coli* with a CMC activity of 0.166 U/mg (Valencia et al., 2013). Unlike the previous Coleoptera species, *T. castaneum*, provided TcEG-1 which has a lower activity compared to other beetle species, but was active at very alkaline pHs (pH 9-11) in both S2 insect cells and yeast (Willis et al., 2011; Shirley et al., 2014). The Coleopteran cellulases were active at 45-50°C similarly to that of mild-thermophilic microbial cellulases.

Two additional insect orders have received attention for their CWD enzymes: Lepidoptera and Orthoptera. *Spodoptera frugiperda'*s larval stage was used as the source of a β-glucosidase BG activity of 2.4 mM^−1^s^−1^ pNPG substrate and was effective when produced in *E. coli* (Marana et al., 2004). Orthopteran species are known for their mandibular action for chewing through fibrous plant tissue. The symbiont *Klebsiella* sp. in the gut from an undisclosed grasshopper species was found to have BG with similar activity of 2.61 ± 0.75 mM^−1^s^−1^ on pNPG to that of *S. frugiperda* (Shi et al., 2011). From *Teleogryllus emma*, an EG from GH family nine was produced in Sf9 cells showing an activity rate of 3118.4 (U/mg) against CMC (Kim et al., 2008).

Insect GHs have not yet been synthesized in transgenic plants. Under heterologous expression in microbial systems, the majority of insect derived GHs demonstrate similar thermal and acidic pH ranges. A few notable examples from Coleoptera have lower activity, but a uniquely alkaline pH range. Attributes, such as high alkaline affinity, learned from insect derived GHs could be utilized in site-directed mutagenesis studies to enhance non-insect biocatalyst research. Overall plant produced insect GHs are another viable avenue for an autohydrolytic plant systems.

## Strategies for engineering autohydrolysis in plants

Constitutive gain-of-function hydrolase activity could have negative phenotypic effects in transgenic plants. While these negative growth effects seldom have been observed in plants harboring GHs with high temperature optima, we envisage host off-effects as a potential issue in heterologous production systems for non-thermophilic enzymes. Use of inducible promoters to attenuate hydrolase activity can reduce off-target effects and decrease chances of lethality (Schena et al., 1991; Taylor et al., 2008). Inducible systems could be utilized at several life cycle/growth stages to specifically induce the hydrolase activity to detect onset of different genes and pathways involved in cell wall biosynthesis. One such inducible approach has been used to control CWD transgene expression in tobacco. An ethanol inducible promoter demonstrated effective cellulase production while preventing dwarfism when compared to constitutively produced cellulase in tobacco plants (Klose et al., 2013).

Protein modification can be used on GHs to control for off-target effects and increase protein yield. Sequestration of proteins by signal peptides to organelles, such as vacuoles, ER, or plastids improves production rate of transgenic proteins, but also isolates them from their intended substrates (Hood et al., 2007; Taylor et al., 2008). The use of hyperthermic proteins, where enzyme activation temperature is above that required for plant growth are useful during biorefinery steps to prevent undesired effects (Mir et al., 2014). A unique protein sequence known as an intein, can be inserted into the portions of enzymes and prevents proper protein folding. Inteins are heat labile and have been proven to successfully prevent undesired enzymatic action until placed in high temperature, which induces intein excision (Shen et al., 2012).

Plants are attractive bioreactors for many recombinant proteins because their post-translational modification systems are analogous to other eukaryotes (Hellwig et al., 2004; Fischer et al., 2012; Zhang, 2015). Plant cell culture systems could also speed the screening process for functional GH production *in planta* prior to generation of time consuming whole transgenic plants (Hellwig et al., 2004; Shen et al., 2013). A multi-well system using a plant cell system for rapid GH stacking of multiple constructs would speed the screening process of GHs.

A current research problem is the variety of substrates used to measure activity and efficiency. Filter paper is reformed cellulose, but the different densities and size of the filter paper disc used during assays have effects on enzyme activity (Xiao et al., 2004). Carboxymethylcellulose is the most common substrate used because of its solubility in liquids, which is caused by methylating cellulose (Reese et al., 1950). Avicel (powdered crystalline cellulose) is a close relative to cellulose, but can be purchased at crystalline particle size making comparisons with the soluble form difficult (Yeh et al., 2010). Both of these (and other cellulose derivative) substrates can also be affixed with fluorophores which are activated upon hydrolysis (Megazyme Wicklow, Ireland). The units differ based on the method used to measure, some do a straight absorbance compared to a control, a reduction in viscosity of a solution, use of a standard curve, or percent of a commercial cellulase standard (Taylor et al., 2008; Willis et al., 2010).

An important decision to be made when developing commercial-ready autohydrolytic plants is the host plant. This issue is important because C4 grasses (leading biofuel crops) have different cell wall composition and genetic mechanisms than C3 plants. While current monocot model species, such as rice (*O. sativa*) and Brachypodium (*Brachypodium distachyon*), have extensive genomics toolsets; e.g., completed genomes and activation tagging systems; both are C3 grasses that are distantly related to C4 grasses (Jeon and An, 2001; Ito et al., 2005; Matsumoto et al., 2005; Vogel et al., 2009, 2010). Thus, while rice and Brachypodium have proven to be powerful tools, they are suboptimal to dissect C4 grass metabolism and cell wall biosynthesis (Dal'Molin et al., 2010). This latter point is a crucial consideration for testing autohydrolytic biofuel crop lines. C4 grasses (e.g., maize, sorghum, and switchgrass) possess divergent expression patterns of cell wall-related genes, and indeed likely use different genes, compared to C3 grasses. The specialized vascular anatomy of C4 plants relies on similar genes to C3 grasses, however, their regulation and expression differ, resulting in different structures; their secondary cell walls are simply not well modeled by C3 grasses (Nelson, 2011). The brown midrib (BMR) mutant phenotype in maize, sorghum, and pearl millet is a classic lignin alteration in cell walls, but no C3 plants have been reported with this cell wall phenotype. Putative BMR ortholog mutants in C3 plants seem to have a different basis; BMR rice mutant orthologs are known, but present different phenotypes if altered (Sattler et al., 2010). A digestibility assay comparing two C3 and two C4 grasses native to Australia showed that C3 grass leaves had more facile enzymatic hydrolysis compared to that of the C4 grasses, which was attributed to C3/C4 differences in leaf anatomy (Wilson and Hacker, 1987). *In vitro* evaluations using purified cellulose and lignin polymer models demonstrated that for lignin to affect cellulose saccharification it must be specifically cross linked to cellulose (Jung et al., 2011). Grass arabinoxylans cross link to lignin by ether bonds using ferulate and diferulate molecules (Burr and Fry, 2009). Dicot cell walls lack arabinoxylans for cross linking to lignin and therefore are inadequate to use as an improved digestibility model for C4 grass crops. Alternatively, dicot cell walls are primarily composed of xyloglucan hemicellulose, which cross link to lignin (Scheller and Ulvskov, 2010; Furtado et al., 2014; Damm et al., 2016). Utilization of C4 grass species is more appropriate for evaluation of CWD enzymes if the target feedstock is a C4 grass. The applied approach for evaluation of novel autohydrolytic lines should be used with recently improved transformation strategies of many C4 crops (e.g., maize, switchgrass, sorghum) to reduce variability to real world marketable biomass (Ishida et al., 2007; Brutnell et al., 2010, 2015; Li and Qu, 2011; Liu and Godwin, 2012; Lambertz et al., 2014).

## Conclusion

There are many economic pinch points in the production of cellulosic biofuels. Enzyme cost is one of them and is not trivial. Using recombinant systems, including the plant feedstock itself, is likely necessary to produce advanced biofuels in a sustainable manner (Gressel, 2008). Plants, including a few species that are relevant proxies for cellulosic feedstocks, have been used as hosts for overexpression of microbial GH genes. We envisage insect-derived GHs playing a role in reducing cell wall recalcitrance in transgenic plants owing to their high diversity of function and eukaryotic origin. Utilization of multiple enzyme classes should be used in concert in feedstocks, coupled with appropriate subcellular targeting as well as spatial and temporal control of synthesis. While there is much research needed to produce a commercial cellulosic feedstock with autocatalytic properties, field experiments with E1-producing transgenic maize demonstrates that research in this area should be worthwhile. The combination of improved biomass feedstocks and enzyme technologies is a step toward a renewable plant-based fuel system.

## Author contributions

JW drafted the manuscript and MM and CS participated in the drafts and revisions. All authors read and consented to the final version of the manuscript.

### Conflict of interest statement

The authors declare that the research was conducted in the absence of any commercial or financial relationships that could be construed as a potential conflict of interest.

## Abbreviations

AU: absorbance unit
AX: arabinoxoylan
AZCL-xylan: azo-crosslinked-xylan
AZCL: azo-crosslinked
BG: β-glucosidase
BMCC: bacterial microcrystalline cellulose
*CaMV35S*: cauliflower mosaic virus 35S promoter
CBH: cellobiohydrolase
CBM: carbohydrate binding module
CMC: carboxymethylcellulose
*CMVar*: cucumber mosaic virus advanced replicating promoter
CWD: cell wall degrading enzyme
FU: fluorescence units
EG: endo-1,4-β-glucanase
ER: endoplasmic reticulum
GH: glycosyl hydrolase
GHF: glycosyl hydrolase family
*GluB-1*: rice glutelin endosperm promoter
HEPES: 4-(2-hydroxyethyl)-1-piperazineethanesulfonic acid
*Hor2-4*: barley B1 hordein promoter
*Mac*: promoter combination of the Ti plasmid mannopine synthetase and CaMV 35S
MUC: 4-methylumbelliferyl-β-D-cellobioside
MUL: 4-methylumbelliferyl-β-D-lactopyranoside
n.d.: not determined
n.o.: not optimized
n.r.: not reported
pNPG: *p*-nitrophenyl β-D-glucopyranoside
RBB-xylan: remazol brilliant blue R-D-xylan
*RbcS-3C*: Rubisco small sub-unit promoter
*TRBO*: tobacco mosaic virus RNA-based overexpression vector
U: unit
Xyl: xylanase
*Zm-PEPc*: maize phosphoenolpyruvate carboxylase.
